# Improving Whitefly Management

**DOI:** 10.3390/insects12050470

**Published:** 2021-05-19

**Authors:** Alvin M. Simmons, David G. Riley

**Affiliations:** 1 U.S. Vegetable Laboratory, Agricultural Research Service, United States Department of Agriculture, 2700 Savannah Highway, Charleston, SC 29414, USA; 2 Department of Entomology, University of Georgia, Bldg 4603, 110 Research Way, Tifton, GA 31794, USA

## Introduction

Whiteflies (Hemiptera: Aleyrodidae), especially the sweetpotato or cotton whitefly, *Bemisia tabaci* (Gennadius), are among the most destructive and difficult to manage polyphagous insect pests around the globe. Because of their global pest status in numerous crops, most of the recent and current whitefly research focuses on members of the *B. tabaci* cryptic species complex. In addition to some of these whiteflies excelling in the development of insecticide resistance [1], their complex relationships with multiple cropping systems and natural enemies [2], and their associations with numerous species of plant viruses [3] make it challenging to design integrated pest management (IPM) programs for consistent crop protection against these whiteflies and the plant viruses that they transmit. However, there have been successful areawide programs that integrate biological control of whiteflies in cotton [4], where the transmission of plant viruses has not been an overwhelming issue. Unfortunately, within many crop systems, whiteflies transmit multiple viruses (about 90% are begomoviruses, 6% criniviruses, and 4% are closteroviruses, ipomoviruses or carlaviruses [3]). Consequently, this whitefly-virus threat greatly complicates many aspects of management and protection of crops. Whitefly-transmitted viruses often cause much greater harm to the crop than the whitefly itself. Thus, the tolerance for a viruliferous adult whitefly is much lower than for a non-viruliferous adult whitefly, which changes the economic injury level for whiteflies [5], and in turn puts greater pressure on the available control tactics, like insecticides or biological control. Host plant resistance against whitefly-transmitted viruses [6] has been one of the more challenging and effective tactics for mitigating this complicating virus-effect on whitefly management. Integration of host plant resistance and other control tactics may ultimately provide the best solution for both whitefly and whitefly-transmitted virus management [7].

The goal of improving whitefly management, ultimately, is to support sustainable crop production for regional agroecosystems. Achieving this goal can be accomplished through clearly identifying the whitefly problem, i.e., direct damage, virus transmission, etc., for a given cropping system, identifying and developing control tactics, e.g., host plant resistance, chemical control, biological control, RNAi control, etc., and improving whitefly management decision criteria. Once the specific whitefly problem has been defined for a cropping system and an array of available tactics has been identified, deciding when and where to implement specific management-activities becomes the next most important piece of information. The Special Issue of *Insects* about “Improving Whitefly Management” provides insight into the recent activities of whitefly research teams around the globe, specifically on what topics they are focused. A myriad of studies has resulted in information that has led to progress on assorted bio-based and traditional strategies for managing both whiteflies and problems from the viruses that they transmit. These range from identifying the whitefly problem through local and regional surveys, developing new and exciting control tactics through the use of RNAi technology, developing and employing integrated pest management tools, such as natural enemies and plant resistance, and improving simple decision tools, such as rapid insecticide resistance bioassays. The body of information in the Special Issue consists of a combination of original research and review of relevant documentations on whiteflies and their associated viruses. Even as whitefly-virus related crop production problems seem to be accelerating around the world, possibly due in part to global climate change and from the transport of infested plant materials, science is in rapid pursuit of both traditional and cutting-edge solutions for mitigating these problems.

Some of the information from research toward identifying the extent of the whitefly problem that is highlighted in this “Improving Whitefly Management” Special Issue includes the following.

- Crossley and Snyder take a broad, worldwide view for defining the whitefly problem by examining the spatial dispersal of genetic variants of *Bemisia tabaci* [8]. This review provides researchers with the state-of-the-art tools for genetic analysis which can impact all aspects of whitefly management.
- The genetic variability in *Bemisia tabaci* MEAM1 populations within the farmscapes of Georgia, USA was reported to be relatively low by Gautam et al. [9]. This established baseline data for regional management in this region which will aid in management efforts going forward.
- An understanding of the regulatory gene networks of whiteflies can help identify targets for RNA interference control of whiteflies. Therefore, Hassagawa et al. conducted research which revealed a comprehensive microRNA regulatory system in the whitefly *B. tabaci* and this may be involved in virus acquisition and transmission [10].
- Plant viruses can influence the bionomics of whiteflies. Huang et al. reported on the effect of the *Tomato chlorosis virus* on *B. tabaci* reproduction by increasing the expression of vitellogenin [11].
- Understanding the population dynamics of pests provides good perspectives for developing management strategies. Krasse-Sakate et al. provided a review of the population dynamics and distribution of the primary species of whiteflies attacking crops in South America and their associated viruses, and management strategies employed [12].
- Soybean is among the crops damaged by whiteflies (*B. tabaci*) in Brazil. A study by Barros et al. demonstrated proximal sensing as a tool for assessing the infestation of *B. tabaci* populations in the field [13].

Information from research toward identifying whitefly or virus control tactics that is highlighted in this Special Issue includes the following.

- One of the most promoted biological controls for whiteflies is the use of generalist predators either through augmentation or conservation of indigenous populations. Kheirodin et al. conducted a worldwide review of the use of predators to control *Bemisia tabaci*, making the strong argument that biological control needs to be one of the first tactics considered when developing an IPM program for this pest [14].
- Biological control provides an environmentally friendly strategy that can be subjected to numerous abiotic and biotic influences. Wu et al. demonstrated that the performance, under a range of temperatures and strong ultraviolet radiation (UV), of a new strain of an entomopathogenic fungi, *Cordyceps javanica*, that originated from a whitefly epizootic, supports the view that this fungus is a good candidate as a biopesticide [15].
- Plant resistance offers a foundational role in providing relief from the threat of whiteflies. Acylsugars-mediated resistance from a wild species, *Solanum pennellii*, introgressed into tomato, *Solanum lycopersicum* L., was demonstrated by Marchant et al. to negatively impact whiteflies and the incidence of the whiteflies to acquire and transmit the *Tomato yellow leaf curl virus* [16].
- Agarwal et al. identified several genotypes of snap bean (*Phaseolus vulgaris*) and lima bean (*P*. *lunatus*) with resistance against two whitefly-transmitted begomoviruses (*Cucurbit leaf crumple virus* and *Sida golden mosaic Florida virus*) [17].
- Technology that inhibits gene expression is among the new strategies for managing insects and other crop pests. A review article by Shelby et al. explores the short and long-term positives and negatives and other considerations associated with gene silencing through RNA interference-mediated control of the whitefly *B. tabaci* [18].
- Primary and secondary endosymbionts in the cells of whiteflies play important roles of in the lives of whiteflies in the agroecosystem. A review article by Andreason et al. focuses on defining the biological, evolutionary and plant interaction roles of endosymbionts of the cryptic species of *B*. *tabaci* [19].
- From the perspective of integrated pest management, over 50 species of economically important whiteflies were discussed in a review that highlighted next-generation control strategies such as nanotechnology, RNA interference, and genetic modifications of plants for the expression of proteins that adversely affect whiteflies [20].
- One of the most daunting controls of whiteflies is that of contaminated fresh produce that is shipped around the world. Cho et al. provide strong evidence for successful use of electron beam and X-ray radiation for the decontamination of fresh strawberries for export [21].

Finally, information from research toward identifying whitefly management decision criteria that are highlighted in this Special Issue includes the following.

- The toxicological bioassays compared by Sparks et al. quantify the utility and limitation of each technique relative to their ability to assess whitefly efficacy so that the best controls can be recommended [22]. Chemical control is still the number one tactic, but it is also the most prone to resistance selection. These bioassays can mitigate resistance by providing the field-specific data for informed use decisions.
- Li et al. provide an extensive review of whitefly problems in vegetables in the southern United States, its economic impact, and management efforts. This review supports pest managers to decide which of the many tactics would be most available for their cropping system in this region [23].

Although there have been many advancements in providing solutions for the improvement of whitefly management around the globe, much more progress is needed. It is abundantly clear that a single management strategy is not likely to solve the whitefly-virus problem. However, by building on and expanding the scientific knowledge base, a better path will be forged for practical solutions for whitefly-virus relief for the agricultural community.
